# A Novel Prophylaxis Strategy Using Liposomal Vaccine Adjuvant CAF09b Protects against Influenza Virus Disease

**DOI:** 10.3390/ijms23031850

**Published:** 2022-02-06

**Authors:** Julie Zimmermann, Signe Tandrup Schmidt, Ramona Trebbien, Rebecca Jane Cox, Fan Zhou, Frank Follmann, Gabriel Kristian Pedersen, Dennis Christensen

**Affiliations:** 1 Center for Vaccine Research, Statens Serum Institut, 2300 Copenhagen S, Denmark; juzi@ssi.dk (J.Z.); sxs@ssi.dk (S.T.S.); frf@ssi.dk (F.F.); gakp@ssi.dk (G.K.P.); 2 Department of Virus and Microbiological Special Diagnostics, Statens Serum Institut, 2300 Copenhagen S, Denmark; ratr@ssi.dk; 3 Influenza Centre, Department of Clinical Science, University of Bergen, 5020 Bergen, Norway; rebecca.cox@uib.no (R.J.C.); Fan.Zhou@uib.no (F.Z.)

**Keywords:** liposomal adjuvant, virus prophylactic (treatment), type I IFN, influenza virus

## Abstract

The SARS-CoV-2 pandemic caused a massive health and societal crisis, although the fast development of effective vaccines reduced some of the impact. To prepare for future respiratory virus pandemics, a pan-viral prophylaxis could be used to control the initial virus outbreak in the period prior to vaccine approval. The liposomal vaccine adjuvant CAF^®^09b contains the TLR3 agonist polyinosinic:polycytidylic acid, which induces a type I interferon (IFN-I) response and an antiviral state in the affected tissues. When testing CAF09b liposomes as a potential pan-viral prophylaxis, we observed that intranasal administration of CAF09b liposomes to mice resulted in an influx of innate immune cells into the nose and lungs and upregulation of IFN-I-related gene expression. When CAF09b liposomes were administered prior to challenge with mouse-adapted influenza A/Puerto Rico/8/1934 virus, it protected from severe disease, although the virus was still detectable in the lungs. However, when CAF09b liposomes were administered after influenza challenge, the mice had a similar disease course to controls. In conclusion, CAF09b may be a suitable candidate as a pan-viral prophylactic treatment for epidemic viruses, but must be administered prior to virus exposure to be effective.

## 1. Introduction

Since the beginning of the 21st century, the world has battled a series of major crises caused by viral epidemics, including SARS-CoV-1 and influenza A H5N1, and pandemics H1N1, MERS-CoV, Ebola virus, and the current SARS-CoV-2, which has underscored how vulnerable we are to emerging viral threats. In a breakthrough for mRNA-based vaccines, several vaccines were approved for human use in less than a year after a global pandemic was declared, which is unprecedented and beyond the most optimistic initial hopes [1]. These vaccines are very effective at preventing disease and death caused by the current SARS-CoV-2 strains and are key components to control the pandemic. However, the damage caused by SARS-CoV-2 in the period prior to vaccine approval in terms of human and economic losses emphasizes the need for a ready-to-use tool for immediate control of viral outbreaks.

Most pathogens capable of causing a pandemic have high mutation rates, which results in evolution into different lineages characterized by variable levels of virulence and transmissibility. This can also lead to the emergence of new variants and potentially evasion from vaccine-induced immunity as well as disease-acquired protection [2]. Several mutations in the immunodominant SARS-CoV-2 Spike protein have been identified, which cause continued concern for the efficacy of the currently licensed vaccines [2,3].

There is therefore an unmet global need for a strategy that can be implemented immediately after an outbreak to reduce the risk of infection, improve disease outcomes, and inhibit transmission to other people, without the use of large-scale quarantines, travel restrictions, and social distancing. An optimal strategy would be pan-viral and its efficacy should not be affected by virus homoplasticity. A pan-viral prophylactic strategy against respiratory virus pandemic threats requires protection against a broad and largely unforeseen spectrum of viruses. The innate immune system forms the body’s emergency preparedness against pathogenic threats and has evolved to recognize and react instantly to pathogenic fingerprints, such as viral double-stranded (ds)RNA [4]. For the last 20 years, research has identified the mechanisms behind how the innate immune system reacts to gain initial control of infections, until the adaptive immune system has had time to form a specific immune response to eliminate the invading pathogen. The innate immune system is therefore in most cases not sufficient to eliminate viral infections with, for example, influenza viruses or coronaviruses, but it can mitigate damaging effects of the virus attack and reduce disease symptoms if properly activated.

One of the key components of the early innate response against viruses is type I interferons (IFN-I). The timing of initiation of an IFN-I response depends on viral and host factors and is critical for the progression of a SARS-CoV-2 infection [5]. Thus, an initial low viral load allows the rapid induction of a strong IFN-I response, which can clear the infection, whereas an initial high viral load will suppress the IFN-I response and cause disease progression [5]. Supporting this, in vitro models show that pretreatment with IFN-α and IFN-β effectively prevents infection with SARS-CoV-2 upon challenge [6]. Furthermore, the IFN-I responses were impaired in SARS-CoV-2-infected patients with severe and critical disease [7]. IFN-I are pleiotropic immunomodulatory cytokines that can activate protective antiviral effects in several cell types, which in concert induce a general antiviral state [8]. Danger signaling is initiated when viral RNA is detected by pattern recognition receptors (PRRs), e.g., the RIG-I and TLR3, 7, and 8 receptors activating IFN-I secretion. However, due to the effective antiviral effects induced by IFN-I, viruses have developed several ways to circumvent an antiviral state, mainly through blocking the expression of IFN-I-related genes [9].

Modern pharmaceutical design and engineering has made it possible to synthetically design TLR agonists and, combined with novel delivery techniques, these can now be safely delivered to mucosal surfaces, which permits clinical testing. In recent clinical trials, intranasal delivery of the TLR3 agonist polyinosinic:polycytidylic acid [poly(I:C)], a synthetic dsRNA structurally similar to virus dsRNA, stimulated IFN-I production and significantly protected against Rhinovirus and Influenza virus infections in humans [10]. This immune prophylaxis was safe and well-tolerated. Infection was less severe, had a shorter duration, and importantly, reduced the number of individuals with study-defined, laboratory-confirmed illness, without compromising seroconversion induced by infection. The novel vaccine adjuvant CAF^®^09b is formulated by electrostatically complexing the TLR3 agonist poly(I:C) with a cationic liposomal delivery system comprising dimethyldioctadecylammonium bromide (DDA) and C-type lectin receptor agonist monomycoloyl glycerol (MMG), and was developed for vaccines against viral infections [11,12] and cancer [13]. CAF09b has thus been combined with numerous vaccine antigens and tested extensively as a vaccine adjuvant preclinically and in humans (NCT03412786, NCT03715985) [14]. There is a risk of adverse effects and toxicity in both humans and animal models when administering unformulated poly(I:C) [15,16]. However, when poly(I:C) is complexed within cationic liposomes, the detrimental innate immune reactions are abrogated [16]. This is because the cationic liposomes retain poly(I:C) at the injection site and thereby prevent systemic distribution of the immunostimulator. The surface charge of CAF09b remains highly cationic after complexation with poly(I:C), which facilitates interaction with and uptake by target cells [13,17]. Therefore, we hypothesize that the cationic liposomes will facilitate local delivery of complexed poly(I:C) to IFN-I-production-capable APCs while minimizing the systemic side effects.

In the present study, we show that intranasal administration of CAF09b to mice caused upregulation of several IFN-I-related genes, and pretreatment with CAF09b prevented death upon lethal challenge with mouse-adapted influenza A A/Puerto Rico/8/1934 (H1N1) (PR8) virus. In contrast, no protective effect was observed when CAF09b treatment was initiated after influenza challenge. Thus, the liposome-based CAF09b is a promising tool for pan-viral prophylaxis against disease and death caused by respiratory viruses with pandemic potential.

## 2. Results

### 2.1. Intranasal Delivery of CAF09b Upregulated IFN-I-Related Genes

The CAF09b liposomes were prepared by the thin-film method, first by formulating the DDA and MMG liposomes by high-shear mixing at a temperature well above the main phase transition temperature of approximately 41 °C [18]. Subsequently, poly(I:C) was associated to the cationic liposomes by slow addition while maintaining the elevated temperature and vigorous agitation. The poly(I:C) must be added as described to avoid heavy aggregation of the liposomes and collapse of the formulation. The properly prepared formulation is highly cationic with zeta potential above 40 mV and particle sizes of 150–200 nm [13,18].

**Figure 1:**
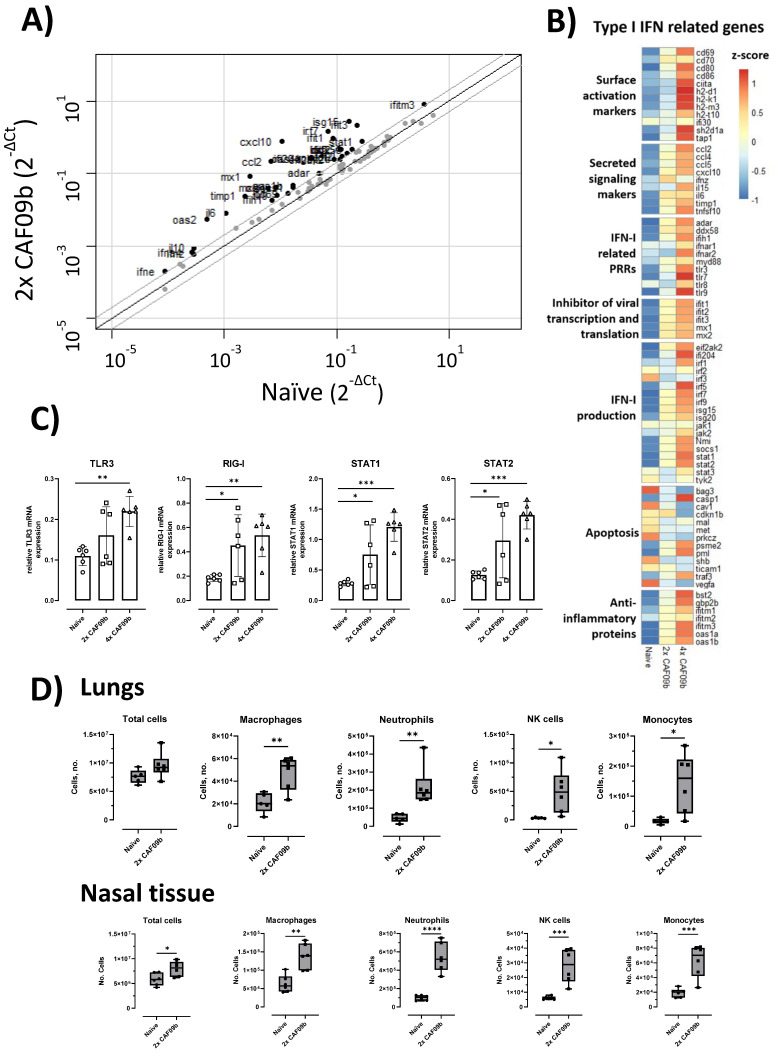
Intranasal administration of CAF09b upregulated IFN-I-related genes and caused an influx of innate immune cells. Mice (CB6F1, *n* = 6) were administered CAF09b 20 µL i.n. on days 0 and 3 (2× CAF09b), or days 0, 1, 2, and 3 (4× CAF09b), the naïve group was administered Tris-buffer 20 µL i.n. on days 0, 1, and 3. (**A**–**C**) Lungs were collected at day 4 and expression of IFN-I related genes were measured by qPCR. (**A**) Scatterplot of the 84 genes related to IFN-I responses. Naïve mice were compared to 2× CAF09b-treated mice. The black line represents a 1-fold change, and the grey lines represent a 2-fold change. Dots represent the average relative mRNA expression for each of the genes. (**B**) Heatmap of an average of the relative mRNA expression for all 84 genes. The three groups are compared against each other and represented by the z-score. (**C**) Plots of the relative mRNA expression value for the four genes TLR3, RIG-I, STAT1, and STAT2. Each dot represents one mouse, boxes denote mean ± S.D. One-way ANOVA followed by Dunnett’s multiple comparisons test with a comparison to the naïve group, * *p*-value ≤ 0.05, ** *p*-value ≤ 0.01, *** *p*-value ≤ 0.001. (**D**) The cellular composition in the lungs and nasal tissue was analyzed on day 4. The total amount of cells in each tissue was counted, while macrophages (F4/80^+^, CD11b^+^), neutrophils (Ly6G^+^), NK cells (NK1.1^+^), monocytes (CD11b^+^, Ly6C^+^), and DCs (MHCII^+^, CD11c^+^) were identified by flow cytometry (Supplementary Figure S1). Box and whisker plots denoting mean and min./max. value, dots represent individual mice. Two-tailed unpaired *t*-test, * *p*-value ≤ 0.05, ** *p*-value ≤ 0.01, *** *p*-value ≤ 0.001, **** *p*-value ≤ 0.0001.

The ability of the CAF09b liposomes to induce IFN-I-related genes was evaluated in a mouse model. CAF09b was administered i.n. twice on days 0 and 3 as well as daily (days 0, 1, 2, and 3). A naïve group was administered Tris-buffer on days 0, 1, and 3. The induction of IFN-I-related genes in the lungs was analyzed by qPCR on day 4. Intranasal administration of CAF09b upregulated several IFN-I-related genes (Figure 1A,B). Out of the 84 IFN-I-related genes assayed, 33 and 42 genes were more than 2-fold upregulated compared to naïve mice after administration of 2 and 4 doses of CAF09b, respectively. No genes were downregulated more than 2-fold after administration of CAF09b compared to naïve mice (Figure 1A and Supplementary Figure S2).

Poly(I:C) is a ligand for TLR3 in endosomes [19], and it is generally believed that RIG-I is one of the primary receptors of cytoplasmic dsRNA [20]. TLR3 was significantly upregulated in the group administered four doses of CAF09b compared to in the naïve group, while RIG-I was significantly upregulated after administration of both two and four doses of CAF09b compared to the naïve group (Figure 1C). STAT1 and STAT2 are transcription factors of Interferon Stimulated Genes (ISGs) and key elements in the IFN-I response. Both two and four doses of CAF09b significantly upregulated STAT1 and STAT2 transcription (Figure 1C).

### 2.2. Intranasal Administration of CAF09b Induced Influx of Several Innate Immune Cell Subsets

In addition to the induction of IFN-I observed in the lungs, we evaluated the influx of different innate immune cell subsets into the lungs and nasal tissue, respectively, following i.n. administration of two doses of CAF09b (Figure 1D). The total cell count in the lungs was not significantly increased by administration of CAF09b, while a significant increase in total cells was observed in the nasal tissue. However, an increase in innate immune cell subsets was observed in both organs compared to naïve mice, although of different magnitude. Thus, the levels of macrophage (F4/80^+^, CD11b^+^), neutrophil (Ly6G^+^), NK cell (NK1.1^+^), monocyte (CD11b^+^, Ly6C^+^), and DC (MHCII^+^, CD11c^+^) subsets were all increased in the lungs and nasal tissue of mice after two doses of CAF09b.

### 2.3. Two Doses of CAF09b Protected against Influenza Disease

**Figure 2:**
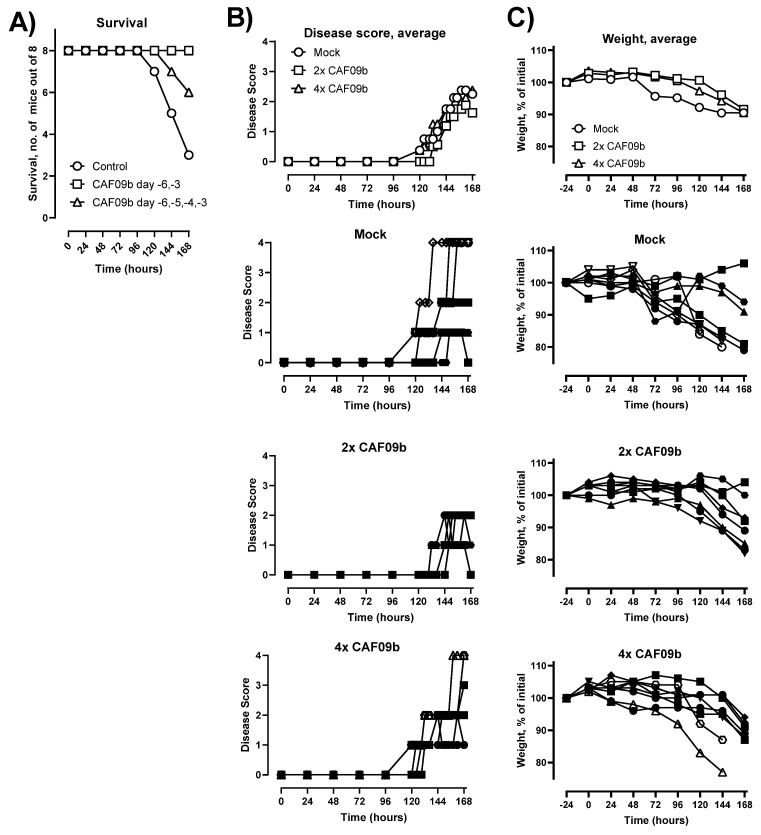
CAF09b i.n. protected against influenza disease. Mice (CB6F1, *n* = 8) were administered CAF09b 20 µL i.n. on days −6 and −3 (2× CAF09b), or days −6, −5, −4, and −3 (4× CAF09b), the mock group was administered Tris-buffer 20 µL i.n. on days −6 and −3. The mice were challenged with mouse-adapted influenza A PR8 H1N1 virus (150 EID_50_, 30 µL i.n.) on day 0. (**A**) Survival curves, mice were euthanized when meeting humane endpoints. (**B**) The disease score was monitored for 168 h after influenza challenge. Euthanized mice were assigned the value 4 for clarity. (**C**) The body weight as a percentage of initial weight (measured on day −1) was measured for 168 h. (**B**,**C**, individual mice): Open symbols: mice were euthanized prior to study termination, closed symbols: mice were euthanized at study termination.

The ability of CAF09b to protect against disease and death caused by lethal influenza challenge was evaluated in the murine model of influenza A H1N1 PR8 virus infection. Two different dosing regimens were tested, and thus CAF09b was administered i.n. twice (on days −6 and −3 prior to challenge) or four times (days −6, −5, −4, and −3). In the mock group, the mice were administered Tris-buffer twice on days −6 and −3. Mice were challenged with mouse-adapted PR8 influenza (150 EID_50_, 30 µL i.n.) on day 0 and their survival was monitored for 7 days post-influenza challenge (p.i.c.) (Figure 2). All mice administered two doses of CAF09b survived in the study, whereas 6/8 and 3/8 mice survived in the groups administered four doses of CAF09b or Tris-buffer, respectively. Thus, two doses of intranasal CAF09b protected against severe influenza disease and there was no additional benefit on disease outcome from administering more CAF09b doses. It was therefore decided to continue the studies with two CAF09b administrations.

### 2.4. CAF09b Was Only Effective When Administered before Influenza Challenge

**Figure 3:**
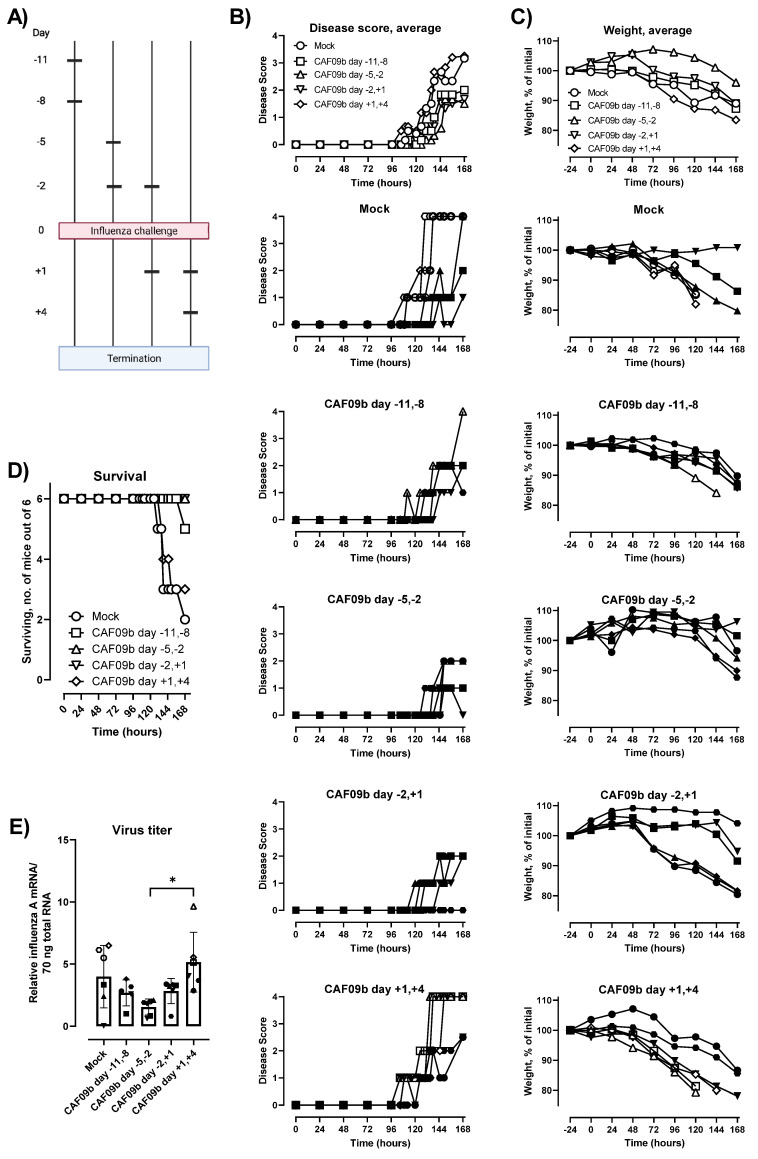
The timing of CAF09b administrations affected protection against influenza virus challenge. (**A**) Mice (CB6F1, *n* = 6) were administered CAF09b 20 µL i.n. twice on days −11 and −8, −5 and −2, −2 and +1, or +1 and +4, and PR8 influenza virus challenge (150 EID_50_, 30 µL i.n.) on day 0. Tris-buffer 20 µL administered i.n. on days −5 and −2 was used as a negative control (mock). (**B**) The disease score was monitored for 168 h after influenza challenge. Euthanized mice were assigned the value 4 for clarity. (**C**) The body weight as a percentage of initial weight (measured on day −1) was measured for 168 h. (**D**) Survival curves, mice were euthanized when meeting humane endpoints. (**E**) The PR8 virus titer was determined in the lungs at the point of euthanization by qPCR, boxes denote mean ± S.D., dots represent individual mice. One-way ANOVA followed by Tukey’s multiple comparisons test, * *p*-value ≤ 0.05. (**B**,**C**,**E**, individual mice): Open symbols: mice were euthanized prior to study termination, closed symbols: mice were euthanized at study termination. (**A**) was created with Biorender.com (Toronto, ON, Canada).

In a pandemic scenario using CAF09b as viral prophylaxis, the exact period between administration of the adjuvant and encountering the pathogen would be unknown. Therefore, we evaluated the effect of CAF09b on preventing disease and death after influenza challenge at different periods around CAF09b administration. Thus, two doses of CAF09b were administered i.n. on days −11 and −8 before influenza challenge (b.i.c.), −5 and −2 b.i.c., −2 b.i.c. and +1 p.i.c., and +1 and +4 p.i.c. A mock group was administered Tris-buffer twice on days −5 and −2 b.i.c. (Figure 3A). The disease severity score, body weight, and survival were evaluated over 7 days p.i.c. (Figure 3B,C). For the groups treated with CAF09b prior to influenza challenge, disease symptoms were reduced but not completely prevented and the onset of disease, measured as an increase in disease severity score, occurred later than for the mock group (Figure 3B,C). In contrast, the disease symptoms and weight curves of the mice starting treatment after influenza challenge were similar to the mock group.

The survival after influenza challenge correlated with the disease severity scores and rate of weight loss. Thus, 6/6 or 5/6 mice survived the study when CAF09b treatment was initiated prior to influenza challenge (Figure 3D). In contrast, 2/6 mice survived in the mock group and 3/6 mice survived when CAF09b treatment started after influenza challenge. CAF09b treatment should therefore be initiated prior to influenza virus infection to alleviate disease and improve survival. The virus titers in mice at the termination of the study were similar across the groups irrespective of CAF09b treatment, although there was a tendency towards lower influenza titers in the day −5, −2 b.i.c. group (Figure 3E).

**Figure 4:**
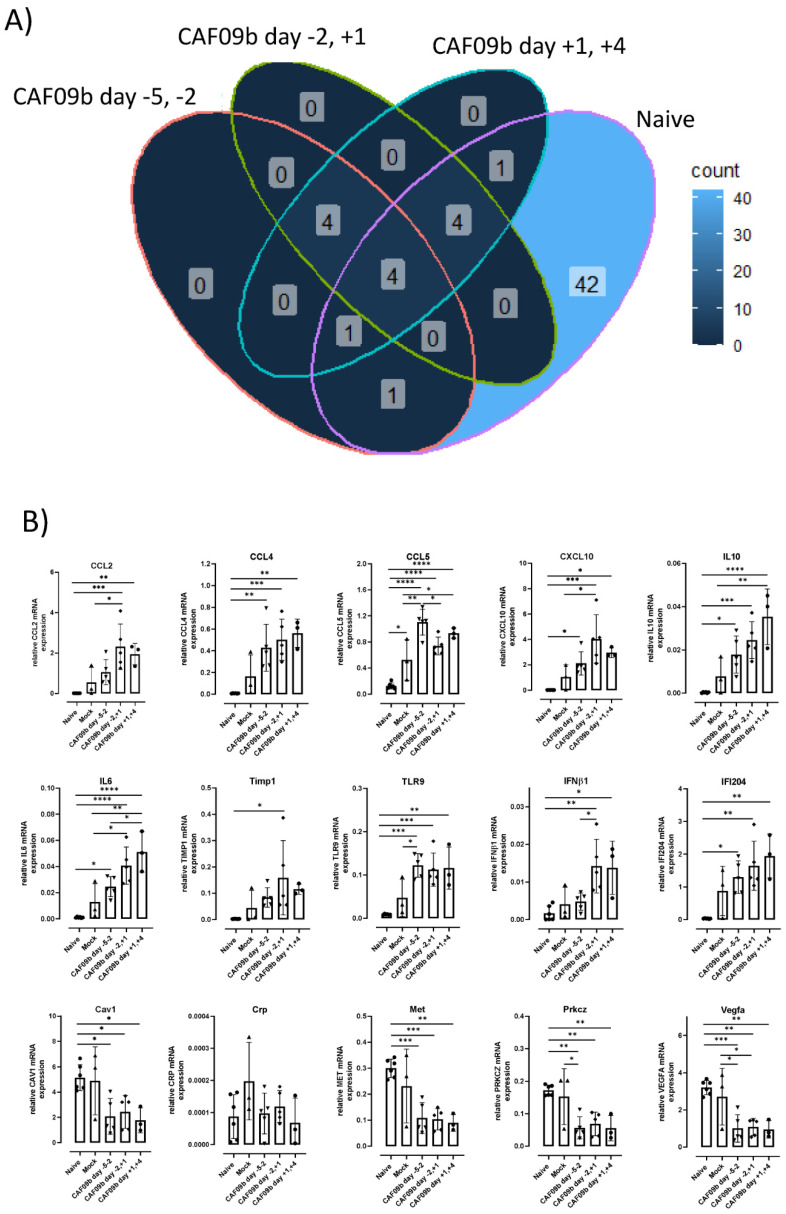
Timing of CAF09b administration minimally affected infection-induced gene expression. Mice (CB6F1, *n* = 3–6) were administered 20 µL CAF09b i.n. twice on days −5 and −2, −2 and +1, and +1 and +4, and challenged with PR8 influenza virus (150 EID_50_, 30 µL i.n.) on day 0. A naïve and a mock group were administered 20 µL Tris-buffer i.n. on days −5 and −2, and the mock group was challenged with PR8 influenza virus (150 EID_50_, 30 µL i.n.) on day 0. Lungs were collected 7 days post-virus challenge and 84 genes related to IFN-I responses were measured by qPCR (lungs were taken at day −1 in the naïve group). (**A**) The Venn diagram shows the number of genes that are more than 2-fold up- or down-regulated compared to the mock group for the four groups (CAF09b days −5 and −2, CAF09b days −2 and +1, CAF09b days +1 and +4, naïve (Supplementary Figure S3)). (**B**) Plots of the relative mRNA expression value of the genes where the mean value is more than 2-fold up- or down-regulated in (**A**). Each dot represents one mouse, boxes denote mean ± S.D. One-way ANOVA followed by Tukey’s multiple comparisons test, * *p*-value ≤ 0.05, ** *p*-value ≤ 0.01, *** *p*-value ≤ 0.001, **** *p*-value ≤ 0.0001.

The expression of genes related to IFN-I responses was measured 7 days p.i.c. in mice administered CAF09b at days −5 and −2 b.i.c., −2 b.i.c. and +1 p.i.c., and +1 and +4 p.i.c., as well as the mock group and the naïve group (Figure 4). The influenza infection highly influenced the expression of IFN-I-related genes. Out of the 84 IFN-I-related genes measured, 53 genes were more than 2-fold up- or down-regulated when comparing the naïve group to the mock group. Furthermore, 42 of these genes were only different in the naïve group. When comparing the CAF09b-treated groups to the mock group, 10–14 genes were more than 2-fold up- or down-regulated (Figure 4A and Supplementary Figure S3). Focusing on the 15 highly up- or down-regulated genes, there was a similar expression profile among the groups treated with CAF09b. CCL2, CCL5, CXCL10, IL10, IL6, and TLR9 gene expressions were significantly higher in some of the CAF09b-treated groups compared to the mock group. In contrast CAV1, MET, PRKCZ, and VEGFA gene expressions were lower in the CAF09b-treated groups compared to the mock group (Figure 4B).

### 2.5. Treatment with CAF09b Did Not Prevent Induction of an Influenza-Specific Antibody Response

**Figure 5:**
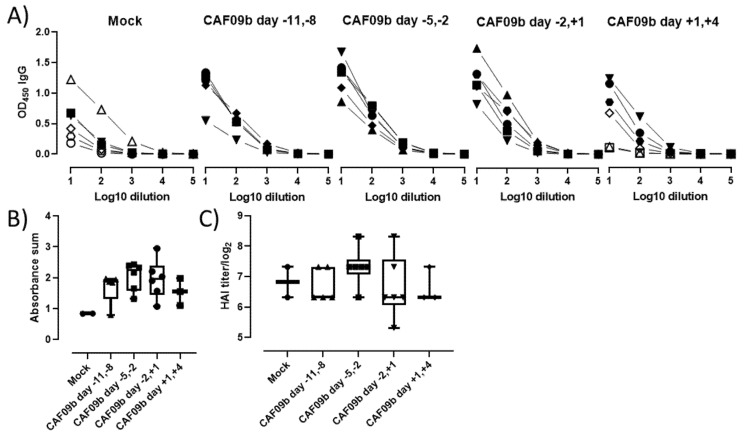
CAF09b did not interfere with induction of antibody responses. Mice (CB6F1, n = 6) were administered 20 µL CAF09b i.n. twice on days −11 and −8, −5 and −2, −2 and +1, and +1 and +4, and challenged with influenza A PR8 H1N1 virus (150 EID_50_, 30 µL i.n.) on day 0. Tris-buffer 20 µL administered i.n. on days −5 and −2 was used as a control (mock). (**A**) The PR8-specific total IgG antibodies in serum were determined by ELISA upon euthanization when reaching a humane endpoint (open symbols) or at study termination 7 days after influenza challenge (closed symbols). (**B**) The sum of absorbance for the individual mice. Only mice which were euthanized upon study termination are included. Box and whisker plots denoting mean and min./max. value, dots represent individual mice. (**C**) HAI titer for individual mice. Only mice which were euthanized upon study termination are included. Box and whisker plots denoting mean and min./max. value, dots represent individual mice.

Induction of an adaptive pathogen-specific memory immune response after infection is critical for protecting the individual from reinfection. To assess if CAF09b treatment interfered with the induction of antibody responses, the levels of PR8 H1N1-specific total IgG antibodies were determined in the blood of the mice at the termination of the study or upon euthanization (Figure 5A,B). All mice in the study developed PR8 H1N1-specific IgG antibodies, indicating that administration of CAF09b did not prevent the induction of adaptive immune responses, despite reducing disease severity. Furthermore, the HAI titers were similar across the treatment groups, indicating that the induced antibody responses were functional (Figure 5C).

## 3. Discussion

Pan-viral prophylaxis could be an effective first-line measure to prevent or reduce the impact of a potential viral epidemic or pandemic by providing a readily available, pathogen-nonspecific treatment. Early after identification of a novel virus with epidemic potential, stimulators of innate immunity which effectively induce antiviral responses could be applied to front-line healthcare workers or close contacts, such as household members of infected individuals. We demonstrated here that CAF09b liposomes could be used to alleviate and prevent influenza-induced disease and death.

Airway administration of CAF09b recruited innate immune cells and robustly upregulated IFN-I-associated genes in the lungs, which together may contribute to the reduction in disease symptoms and prevention of death upon a subsequent influenza infection. The influx of innate immune cells is likely due to the cationic charge of CAF09b, as very similar influx patterns have been observed following intraperitoneal administration of CAF09b and CAF04, a similar adjuvant without poly(I:C) (unpublished data, manuscript in preparation). The cationic nature of the liposomes causes a local inflammatory response [21], which in turn recruits innate immune cells. The inflammatory response and innate cell recruitment induced by CAF09b may be due to similar mechanisms as observed for other cationic particles, which induce necrosis of target cells by interaction with Na^+^/K^+^-ATPase and thereby danger-associated molecular pattern (DAMP) signaling [21]. The cell populations recruited to the nose and lungs after CAF09b administration may have both beneficial and detrimental effects on the antiviral response. Thus, neutrophils may exert antiviral effects, e.g., by secreting antiviral agents such as reactive oxygen species and α-defensins, but may also have damaging effects by promoting a prolonged inflammatory response at the site of infection [22]. The role of NK cells in viral infections is not fully understood, but they are recruited in large numbers by different viruses and may contribute to protection both via direct cytotoxicity and via inducing an antiviral state [23]. However, the cationic charge of DDA-based liposomes, such as the CAFs, does not only exert their adjuvant effect by inducing inflammatory responses. The liposomes are preferentially endocytosed by APCs at the injection site and in the spleen in an energy-dependent manner [17,18]. DDA-based liposomes were further shown to enhance the cellular uptake of associated antigen [17], and it is expected that the cellular uptake of poly(I:C) delivered in CAF09b is similarly enhanced. Since DCs and macrophages are the main producers of IFN-Is, CAF09b was hypothesized to be a good candidate for delivering poly(I:C) to the optimal cell subsets.

Early IFN-I responses are critical to prevent disease due to virus infections and prophylactic or therapeutic antiviral strategies aiming to induce IFN-I after intranasal administration have been evaluated in clinical trials. Two PrEP-001 human clinical trials administering powdered poly(I:C) i.n. twice, 48 and 24 h prior to challenge with either rhinovirus or influenza A virus, showed reduced development of clinical illness and symptoms [10]. In another study, IFN-β-1a was i.n. dosed once daily up to 14 days to patients hospitalized with COVID-19 symptoms, which was well-tolerated and resulted in greater chances of recovery compared to a placebo group [24]. Supporting these findings, a similar clinical study administering IFN-α-2b i.n. to patients admitted to hospital with COVID-19 reduced pro-inflammatory cytokine levels and improved the recovery rate compared to treatment with the antiviral agent arbidol hydrochloride [25]. However, the timing of direct IFN-I (-α or –β) administration has to be considered, as there is a concern of exacerbation of the cytokine storm observed in later stages of severe disease [24]. Indeed, abrogation of IFN-I responses in *Ifnar^−/−^* Balb/c mice resulted in milder disease after SARS-CoV infection compared to wildtype Balb/c mice [26]. Furthermore, disease-delayed IFN-I caused inflammation and abrogated the antigen-specific T-cell responses [26].

The importance of an early IFN-I response was demonstrated in studies with the SARS-CoV-1 virus. Here, animal studies showed that even in complete absence of T- and B-cells, animals were able to control the infection if the innate immune system was alert and able to instantly produce IFN-I after infection [27,28]. These studies also showed that the early innate immune responses could facilitate stronger adaptive immunity to infection. In support of this, both prophylactic and post-exposure strategies involving specific innate immune stimulation, especially via TLR3, have been shown to be able to prevent or eliminate a range of viral infections [29,30].

Importantly, we showed that CAF09b liposomes had to be administered prior to influenza challenge to be effective at preventing symptomatic disease and improving survival. This is in accordance with a study using the poly(I:C) analogue Hiltonol^®^ [poly(ICLC)] as i.n. prophylaxis prior to challenge with mouse-adapted SARS-CoV in Balb/c mice, where treatment had to be initiated within 8 h after virus challenge to prevent disease and death [31]. The requirement for pretreatment with CAF09b indicates that the antiviral environment in the nasal tissue and lungs induced by i.n. administration of CAF09b liposomes must be present at the time of infection. Possibly, virus-induced inhibition of IFN-I responses may hamper the effect of CAF09b when administered post-challenge during, for example, influenza and coronavirus infections, where the innate response is corrupted by the virus [26,32], whereby initial virus growth is allowed without immune pressure. This leads to a delayed immune reaction to the infection and more severe disease.

Elevated levels of several proinflammatory cytokines have been correlated with disease severity across different respiratory virus infections [33,34]. Two identified cytokines, IL-6 and IL-10, were shown to be significantly elevated in mice administered CAF09b after influenza infection (Figure 4B). The cytokines have both beneficial and detrimental effects on the immune responses, which in turn affect disease severity depending on the virus infection (influenza, respiratory syncytial virus, or SARS-CoV-2) [33]. As mentioned earlier, the timing of CAF09b treatment to virus infection may not be known in a clinical setting, and it is therefore necessary to further elucidate any possible exacerbation of existing virus-induced disease caused by CAF09b administration. This may help to determine whether high-risk groups in the human population should be excluded from CAF09b treatment in a clinical setting.

The presented approach may offer a means to tackle the ever-present threat of emerging respiratory viruses with pandemic potential, by offering a strategy to delay virus spread or reduce the negative impact in society until vaccine roll-out can be initiated. Future studies will aim at testing the longevity of protection against different viruses.

## 4. Materials and Methods

### 4.1. Preparation of CAF^®^09b

Dimethyldioctadecylammonium bromide (DDA) and monomycoloyl glycerol [16] were obtained from NCK A/S (Farum, Denmark) and polyinosinic:polycytidylic acid was bought from Dalton Pharma Services (North York, ON, Canada). The liposomal adjuvant CAF09b was essentially prepared as described elsewhere [13]. Briefly, weighed amounts of DDA and MMG were dissolved in EtOH, 96%. A lipid film was formed by evaporating the EtOH under a gentle N_2_ stream for 2 h followed by air-drying overnight. The lipid film was rehydrated in Tris-buffer (10 mM, pH 7.0) with 2% *w*/*v* glycerol by high-shear mixing by using a Heidolph Silent Crusher equipped with a 6F shearing tool (Heidolph Instruments GmbH, Schwabach, Germany) at 26,000 rpm and 60 °C for 15 min. Poly(I:C) was added continuously during high-shear mixing using a peristaltic pump (Pharmacia Biotech, Stockholm, Sweden) over 30 min. The final CAF09b dose was 250/50/12.5 µg DDA/MMG/poly(I:C) in 20 µL.

### 4.2. In Vivo Studies

The induction of innate immune cell responses and prevention of influenza disease after intranasal administration of CAF09b was evaluated in vivo in CB6F1 mice (BALB/c × C57BL/6, Envigo, Horst, The Netherlands). The animal experiments were conducted in accordance with EU directive 2010/63/EU and regulations set forth by the Danish National Committee for the Protection of Animals used for Scientific Purposes. The mice were randomized in the study groups (*n* = 6 or 8) and allowed free access to food, water, and recreational stimuli.

The mice were treated with 20 µL of CAF09b i.n. or Tris-buffer (10 mM, pH 7.0) as a negative control at different time points prior to termination (the day after the last treatment) or to challenge with 30 µL of influenza A Mouse-adapted influenza virus strain A/Puerto Rico/8/1934 virus (5 × 10^3^ EID_50_/mL administered as 15 µL/nostril). The virus was propagated in the allantoic cavity of 10-day-old embryonate hen’s eggs. Allantoic fluid was harvested, clarified, and frozen at −80 °C until use. In the influenza virus challenge studies, the mice were followed for 7 days after challenge and monitored for changes in weight and disease score. The disease score was assessed by trained animal caretakers using an in-house standard protocol (score 0: not affected—normal behavior; score 1: slightly affected—slower movements and maybe slight piloerection; score 2: affected—sitting still but moving when cage is agitated, maybe piloerection, slightly changed respiration, slightly squinting, arching abdomen, hunchback; score 3: clearly affected—move only when prodded, maybe piloerection, labored respiration, half-closed eyes, arching abdomen, hunchbacked; score 4: very affected—slight movement only when prodded, maybe piloerection, labored respiration, closed eyes, cool). The mice were euthanized during the course of the study if they met the predefined humane endpoints: weight loss exceeding 20% of initial weight, and a disease severity score of 2 for more than 48 h or 3 for more than 12 h. At termination, one lung was removed into RNAlater (Thermo Fisher Scientific, Waltham, MA, USA) for evaluation of induction of IFN-I, and the other lung was removed into RPMI 1640 (Gibco, Invitrogen, Carlsbad, CA, USA) for assaying the infectious influenza virus titer. Blood was collected for evaluation of PR8 H1N1-specific antibody titers. In the study terminated prior to influenza virus challenge, the mice were administered anti-CD45:FITC intravenously 3 min prior to euthanization for staining of blood leukocytes. After euthanization, the nasal tissue (upper jaw and nose in front of the eyes) and lungs were removed into RPMI 1640 for identification of the innate cell responses.

### 4.3. Innate Immune Cell Characterization by Flow Cytometry

For evaluation of the innate cell response in the lungs and noses after treatment with CAF09b, the lungs and noses were processed to obtain single-cell suspensions. Each lung was immersed in 2.5 mL of cRPMI (RPMI 1640 supplemented with 5 × 10^−6^ M 2-mercaptoethanol, 1% pyruvate, 1% HEPES, 1% (*v*/*v*) premixed penicillin-streptomycin solution (Invitrogen Life Technologies, Invitrogen, Carlsbad, CA, USA), 1 mM glutamine, and 10% (*v*/*v*) fetal calf serum (FCS)) with 1.6 mg collagenase (Sigma-Aldrich, St. Louis, MO, USA) and processed twice on a GentleMacs using the lung program (Miltenyi Biotec, Köln, Germany), with 30 min incubation at 37 °C in between. The homogenate was then passed through a 100 µm nylon mesh cell strainer (Corning Inc., Corning, NY, USA) and washed twice in cold PBS (Gibco). The noses were cut into smaller pieces prior to incubation with 1.6 mg of collagenase in 2.5 mL of cRPMI+10% FCS for 30 min at 37 °C with agitation. The detached cells were then passed through a cell strainer and washed twice in cold PBS.

The single-cell suspensions were placed in 96-well V-bottomed plates at 10^6^ cells/well, treated with Fc-block, and stained with live-dead cell marker:AF488, CD19:FITC, Ly6G:PE, CD49d:PerCP-Cy5.5, CD11b:PE-Cy7, F4/80:APC, Ly6C:APC-Cy7, NK1.1:BV421, CD11c:BV510, and MHC II (IA-IE):BV605 (eBiosciences, San Diego, CA, USA or BD Biosciences, San Jose, CA, USA). The cells were analyzed using a LSRFortessa with FACSDiva software (BD Biosciences, San Jose, CA, USA) and the data were analyzed using FlowJo (BD Biosciences, San Jose, CA, USA). Cells were identified as macrophages (F4/80^+^, CD11b^+^), neutrophils (Ly6G^+^), natural killer (NK) cells (NK1.1^+^), monocytes (CD11b^+^, Ly6C^+^), and dendritic cells (DC) (MHCII^+^, CD11c^+^), and the gating strategy is shown in Supplementary Figure S1.

### 4.4. Type I IFN Induction by qPCR

Lungs were removed into RNAlater, where they were kept at 4 °C for a minimum of 24 h and then stored at −20 °C. RNA was isolated using the RNeasy mini kit (Qiagen, Hilden, Germany), and lung tissue was homogenized by gentleMACS using the RNA_01 program (Miltenyi Biotec). Genomic DNA was removed by on-column DNase digestion using the RNase-Free DNase set (Qiagen). The quality of the RNA was determined by NanoDrop™ 2000/2000c Spectrophotometers (Thermo Fisher Scientific, Waltham, MA, USA) and the 2100 Bioanalyzer (Agilent Technologies, Santa Clara, CA, USA). All samples had a RIN value > 8. The cDNA was synthesized by the RT^2^ First Strand Kit (Qiagen). Quantitative real-time PCR (qRT-PCR) was performed on a LightCycler^®^ 480 (Roche, Basel, Switzerland) using AbsQuant 2nd Derivative Max for obtaining the Ct value. PCR conditions were 10 min at 95 °C followed by 45 two-step cycles of 15 s at 95 °C and 1 min at 60 °C. For RNA profiling, the RT^2^ Profiler Array “Type I Interferon Response” (Cat. No. 330231 PAMM-016ZA) (Qiagen) was used together with RT^2^ SYBR^®^ Green Mastermix (Qiagen,). The relative mRNA amount was obtained by the ΔΔCt method [35], with the use of the three housekeeping genes GAPDH, GUSB, and HSP90AB1.4.5

### 4.5. Virus Titer Determination by qPCR

The virus titers were determined on lung supernatants from infected mice. The lungs were removed into RPMI and homogenized by gentleMACS using the RNA_01 program (Miltenyi Biotec). The lung supernatant was stored at −80 °C, and the RNA was isolated by the Quick-RNA Viral Kit (Zymo Research, Tustin, CA, USA). The quality of the RNA was determined using a NanoDrop™ 2000/2000c Spectrophotometer (Thermo Fisher Scientific). The titers were determined by the virotype Influenza A RT-PCR Kit (Indical Bioscience, Leipzig, Germany), using 70 ng of RNA. The qRT-PCR was performed on a LightCycler^®^ 480 (Roche)using AbsQuant 2nd Derivative Max for obtaining the Ct value. PCR conditions were 10 min at 45 °C, 10 min at 95 °C, followed by 40 cycles of 15 s at 95 °C and 1 min at 60 °C. The relative mRNA amount was obtained by the ΔΔCt method [35], using β-actin as a housekeeping gene.

### 4.6. PR8 H1N1 Hemagglutinin Protein-Specific Antibody ELISA

Serum was obtained after centrifugation of the blood for 10 min at 10,000× *g* and stored at −20 °C until further use. The PR8 H1N1 antigen-specific IgG antibody responses induced by influenza PR8 challenge were evaluated by ELISA. PR8 H1N1 hemagglutinin protein (Sino Biological Inc., Beijing, China), 1 µg/mL, was coated onto MaxiSorp plates (Nunc, Hillerød, Denmark) overnight at 4 °C. Serum was added at 10-fold serial dilutions and incubated for 2 h at room temperature (rt), followed by incubation with HRP-conjugated anti-mouse total IgG antibodies (AH diagnostics, Tilst, Denmark) for 1 h at rt. The signal was detected by TMB (Kem-En-Tec, Taastrup, Denmark) and the reaction stopped with 0.2 M of H_2_SO_4_, followed by analysis on a TECAN Sunrise™ ELISA reader (Tecan Trading AG, Männedorf, Switzerland) at 450 nm with 620 nm correction.

### 4.7. PR8 H1N1 Hemagglutinin Inhibition Assay

The hemagglutinin inhibition (HAI) assay was performed on fresh guinea pig red blood cells (RBC). The PR8 virus titer at four times the lowest titer causing hemagglutination was used in the assay. The serum was treated with receptor-destroying enzyme overnight at 37 °C followed by inactivation at 56 °C for 30 min. The serum was then incubated with RBCs at 4 °C for 1 h followed by centrifugation at 500× *g* for 10 min and collection of the supernatant. The treated serum was 2-fold serial diluted starting with a 10-fold dilution, and diluted PR8 virus was subsequently added and the samples were incubated at rt for 15 min. RBCs at 0.65% were added to all wells and the plate was incubated at rt for 1 h. The HAI titer was determined as the highest serum dilution retaining a RBC pellet after incubation.

### 4.8. Statistical Analysis

Statistical analyses were performed using either GraphPad Prism software version 8.3.0 for Windows (GraphPad Software, La Jolla, CA, USA) or R (version 4.0.2). Statistical significance between multiple groups was determined by one-way ANOVA followed by either Dunnett’s multiple comparisons test (if all groups are compared to the naïve) or Tukey’s multiple comparisons test (if all groups are compared to each other). Statistical significance between two groups was determined by the two-tailed unpaired *t*-test. Two-fold up/downregulated genes were determined in R and plotted in a scatterplot (car package). The difference in relative mRNA expression for each gene was calculated by the z-score and illustrated in a heatmap (pheatmap package).

## 5. Conclusions

These encouraging early preclinical data using the liposome-based vaccine adjuvant CAF09b suggest a possible potential for a prophylaxis strategy against respiratory viral disease involving activation of innate immunity, especially IFN-I responses to establish anti-viral innate immunity against pandemic viruses. While CAF09b does not prevent influenza infection, it may reduce the severity of disease and death caused by the virus. Furthermore, it will have the potential to afford the immune system time to form the necessary adaptive immunity to protect against recurrent infections or even accelerate the development of adaptive immunity among infected individuals.

## 6. Patents

Patent application No. WO 2021/209562, named “Liposomal composition for preventing or early treatment of pathogenic infection”, resulted from the work reported in this manuscript. F.F. and D.C. are co-inventors on the mentioned patent application.

## Data Availability

Data supporting the reported results can be made available upon request to the corresponding author.

## Supplementary Materials

The following supporting information can be downloaded at: https://www.mdpi.com/article/10.3390/ijms23031850/s1.
