# Supplementary material for: A Novel Prophylaxis Strategy Using Liposomal Vaccine Adjuvant CAF09b Protects against Influenza Virus Disease
Source: Int J Mol Sci. 2022 Feb 6;23(3):1850. doi: 10.3390/ijms23031850 (PMC8836410; doi:10.3390/ijms23031850)
Supplement: Supplementary file 1 [file ijms-23-01850-s001.zip › ijms-1535947-supplementary.pdf]

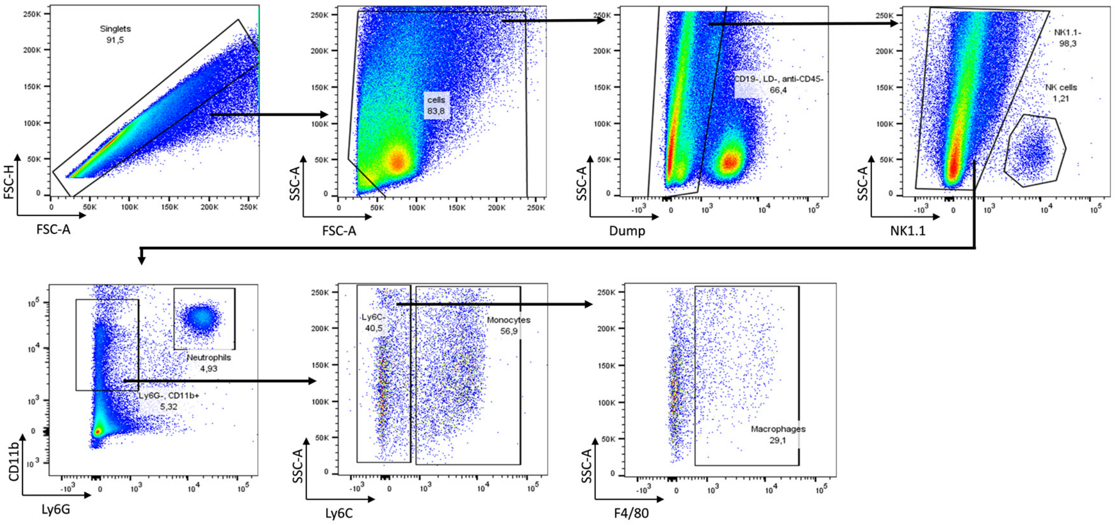

Figure S1. Flow cytometry gating strategy using a lung from a mouse administered CAF09b twice.

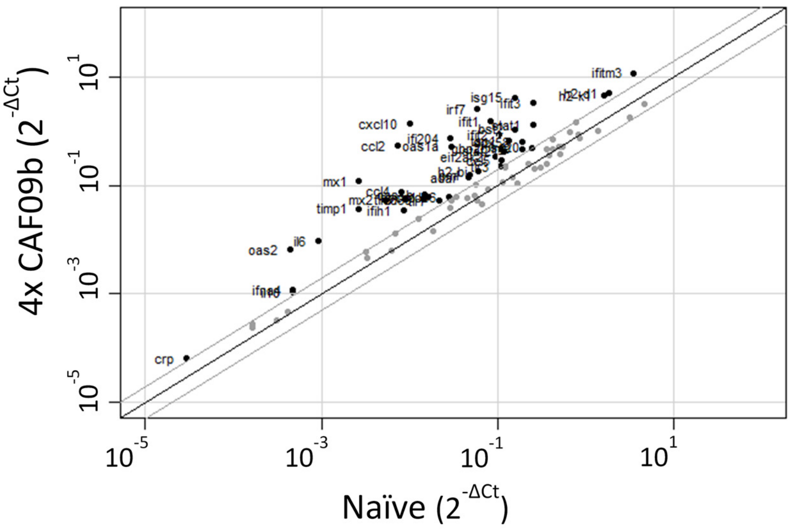

Figure S2. The effect of four times i.n. administration of 20 μL of CAF09b on the type I interferon related genes. Naïve vs 4× CAF09b.

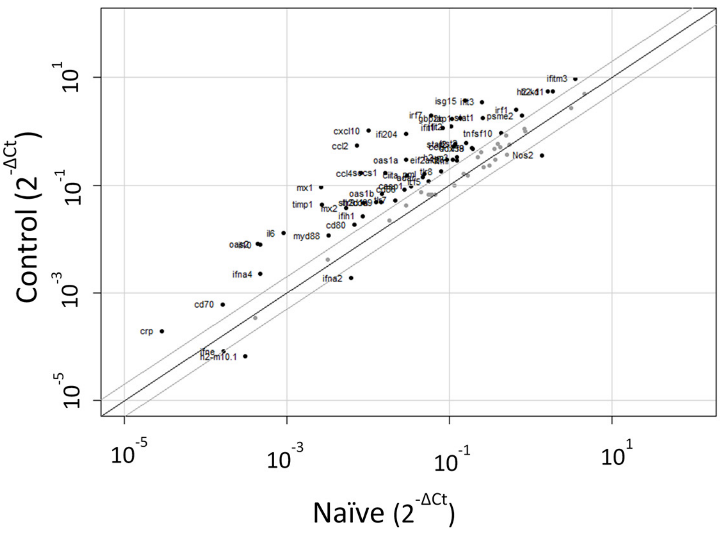

Figure S3. The effect of influenza challenge. Control mice vs. naïve mice.
